# Serum Levels of Chemerin in Patients with Inflammatory Bowel Disease as an Indicator of Anti-TNF Treatment Efficacy

**DOI:** 10.3390/jcm10194615

**Published:** 2021-10-08

**Authors:** Marcin Sochal, Jakub Fichna, Agata Gabryelska, Renata Talar-Wojnarowska, Piotr Białasiewicz, Ewa Małecka-Wojciesko

**Affiliations:** 1 Department of Sleep Medicine and Metabolic Disorders, Medical University of Lodz, 6/8 Mazowiecka Street, 92-215 Lodz, Poland; agata.gabryelska@gmail.com (A.G.); piotr.bialasiewicz@umed.lodz.pl (P.B.); 2 Department of Biochemistry, Medical University of Lodz, 6/8 Mazowiecka Street, 92-215 Lodz, Poland; jfichna@hotmail.com; 3 Department of Digestive Tract Diseases, Medical University of Lodz, 90-153 Lodz, Poland; r-wojnarowska@wp.pl (R.T.-W.); ewuncia@poczta.onet.pl (E.M.-W.)

**Keywords:** chemerin, inflammatory bowel disease, anti-TNF

## Abstract

Chemerin belongs to the adipokines—proteins secreted by white adipose tissue. It plays an important role in angiogenesis and metabolism and its levels correlate with inflammation severity in many clinical states. Circulating chemerin levels in IBD are only rarely evaluated, with inconsistent results. The possible impact of anti-TNF therapy treatment in IBD on chemerin levels has not been addressed. The study aim was to evaluate the serum levels of chemerin in patients with inflammatory bowel disease (IBD), depending on disease severity as well as anti-TNF treatment. Serum chemerin was measured with ELISA in 77 patients with IBD as well as in 42 healthy controls (HCs). Twenty-six participants who underwent anti-TNF therapy were re-examined after 14 weeks. Overall, IBD patients had significantly higher serum chemerin levels than HCs. In patients with IBD exacerbation, chemerin levels were significantly higher compared to the remission group. Serum chemerin levels were significantly higher in UC patients compared to CD. Chemerin correlated with the severity of CD, but not with UC. Serum levels of chemerin decreased significantly after 14 weeks of anti-TNF treatment. Chemerin correlated with the clinical severity of IBD, and its levels decreased after anti-TNF treatment, which suggests its relationship with disease activity. It may be assumed that chemerin levels may possibly be useful for anti-TNF clinical course and treatment monitoring.

## 1. Introduction

Inflammatory bowel disease (IBD) is a chronic immune disease, with two main subtypes: Crohn’s disease (CD) and ulcerative colitis (UC). In recent years, the prevalence of inflammatory bowel disease is rising very rapidly [1].

Adipokines are hormones secreted by white adipose tissue (WAT) [2]. One of these is chemerin, which has a pro- and also anti-inflammatory function, which was first identified in 1997 in keratinocyte and fibroblast cultures [3]. It is synthesized as an inactive prochemerin, which is converted into its active form by serine proteases. Chemerin is secreted in large amounts not only in WAT, but also in the skin, colon, and lungs [4,5]. It binds to three types of receptors: ChemR23, GPR-1, and CCRL2 [6].

High serum levels of chemerin have been found in systemic lupus erythematosus [7], diabetes mellitus [8], asthma [9], esophageal, gastric [10], and colorectal cancer [11]; it correlates with the severity of inflammation in many immune-related diseases—for example in systemic sclerosis or psoriasis [12,13]. It stimulates macrophages to produce pro-inflammatory cytokines, such as tumor necrosis factor (TNF) and interleukin (IL) 6. A study has shown an increase in the expression of ChemR23 receptors in endothelial cells caused by pro-inflammatory cytokines, such as IL 1β, IL-6, and TNF [14].

Most reports point to the pro-inflammatory role of chemerin, but some data show its anti-inflammatory effects. Chemerin reduces neutrophil transepithelial migration, and CHEmR23 activation enhances apical neutrophil clearance [15]. It was also observed that its activation by a chemerin isoform (chemerin 15) in peritoneal macrophages inhibited the production of inflammatory mediators in response to IFN-γ and LPS. Therefore, chemerin may also contribute to acute inflammation suppression or termination [16].

Until now, circulating chemerin in IBD patients was only rarely evaluated and showed inconsistent results [17,18,19,20]. It has been noticed that the clinical condition of patients negatively correlates with body mass index (BMI), which may be due to reduced consumption and absorption of nutrients, as well as increased energy expenditure caused by the disease [21].

Activation of ChemR23 causes the migration of dendritic cells (DCs) to inflammation sites and stimulates phagocytosis [22]. An increased number of these cells in biopsy specimens from patients with active IBD has been observed, which may point to a possible local pro-inflammatory role of chemerin [23]. In another study, it has been shown that the expression, secretion, and processing of chemerin in the cecum and colon are positively associated with the severity of inflammation in dextran sodium sulfate (DSS)-induced colitis. [24]. A breakthrough in the treatment of IBD was the introduction of biological agents mainly based on anti-TNF antibodies, such as infliximab (IFX) or adalimumab (ADA), which are highly efficacious in severe cases [25,26]. These drugs are used in a variety of inflammatory conditions, such as various types of arthritis, hidradenitis suppurativa and psoriasis [27,28]. In rheumatoid arthritis patients, a significant decrease in serum chemerin was observed following anti-TNF therapy [29]. Up to now, no similar studies have been performed in IBD.

Therefore, the aim of the study was to further evaluate serum chemerin in IBD patients, depending on disease severity and anti-TNF therapy.

## 2. Materials and Methods

Seventy-seven patients from the Department of Digestive Diseases in Lodz, Poland were recruited for the study (45 with CD and 32 with UC). The study also included 42 healthy controls (HCs) matched for age, gender, and BMI with the study group.

Among the IBD patients studied, twenty-six patients also underwent anti-TNF therapy (infliximab–IFX or adalimumab–ADA) as a part of the treatment program, following the medicinal products’ characteristics and the insurer’s guidelines. ADA was administered subcutaneously, initially at a dose of 160 mg, then 80 mg in 2nd week, followed by 40 mg every 2 weeks. IFX was administered at a dose of 5 mg/kg body mass intravenously. Subsequent doses were administered at 2, 6, and 14 weeks of therapy. Anti-TNF therapy was received by 20 CD patients and 6 with UC.

To assess the clinical severity of IBD, the Harvey–Bradshaw index (HBI) was used for CD patients and the Partial Mayo Score (PMS) for UC patients. A patient was assigned to the group with IBD relapse if they scored >4 or >1 points, respectively. Information on the course of the disease, time of diagnosis, the presence of perianal fistulas/fissures, other chronic diseases (such as hypertension, diabetes, ischemic heart disease, psoriasis, migraine, endometriosis, arthritis, asthma, primary sclerosing cholangitis), history of abdominal surgery, BMI, and smoking status were obtained. The location of the disease lesions was determined based on the medical documentation, colonoscopy, and gastroscopy, in particular.

A sample of venous blood (4 mL) was collected from each study participant and obtained serum was stored a temperature of −80 °C. Patients treated with anti-TNF therapy were examined and samples of venous blood were collected at two time points: immediately before and after 14 weeks of anti-TNF therapy.

Inclusion criteria contained signed consent to participate in the study, an age of 18–65 years, and a diagnosis of CD or UC. Exclusion criteria were an active malignant neoplastic disease besides basal cell carcinoma and abdominal surgery in the last six months.

The chemerin measurements were performed with ELISA, according to the protocol supplied by the producer (Finetest, Wuhan, China). The serum was diluted 1:100. The detection range of the ELISA kit was: 0.156–10 ng/mL. Additionally, the ratio of the serum level of chemerin before treatment to its concentration after therapy was calculated.

The study was accepted by the Ethical Committee on human research of the Medical University of Lodz, Poland (number: RNN/132/18/KE). The research was conducted ethically following the World Medical Association Declaration of Helsinki. All subjects received all relevant information on the study and were asked to sign informed consent. The study did not affect the inclusion and course of anti-TNF therapy.

Statistical analysis was performed using Statistica 13.1PL (StatSoft, Tulsa, OK, USA). The Shapiro–Wilk test was used to assess the normality of the distribution of the variables. The differences between the variables with normal distribution were measured by *t*-test; between non-normally distributed variables, the Mann–Whitney U test was used. Data are presented as means with a standard deviation, while normally distributed, or as medians with an interquartile range otherwise. The χ2 test was used to compare nominal variables. Differences between dependent variables were assessed by the Wilcoxon test. Correlations were calculated using the Pearson test for normally distributed variables and the Spearman test for non-normally distributed variables.

## 3. Results

Patient characteristics are presented in Table 1.

The patients had the following inflammatory lesion localizations: 53 in the colon (68.8%), 11 in the ileum and colon (14.6%), and 4 in the ileum (10.3%); 3 patients had erosions or ulcers detected at gastroscopy as well as inflammation, erosions or ulcers in the small intestines (3.8%), and 2 had no data (2.5%). No statistical difference was observed between serum chemerin levels based on inflammatory changes location (*p* > 0.05).

Overall, IBD patients had significantly higher chemerin levels than HCs (*p* = 0.047). Among them, only patients with active disease had significantly higher serum levels of chemerin than HCs (*p* = 0.001), which was not observed in patients in remission. Moreover, patients with IBD exacerbation had significantly higher chemerin levels compared to patients in remission (*p* = 0.002, Table 1).

Serum chemerin levels were significantly higher in UC patients (557.4 ± 219.9 ng/mL) compared to CD (446.0 ± 252.6 ng/mL; *p* = 0.048). Mean chemerin levels were significantly higher in UC patients compared to controls (557.4 ± 219.9 ng/mL vs. 404.5 ± 194.2 ng/mL; *p* = 0.002) and not different in CD patients and controls (446.0 ± 252.6 ng/mL vs. 404.5 ± 194.2 ng/mL; *p* = 0.395). Patients with CD exacerbation had significantly higher chemerin levels than those with CD remission (528.1 ± 235.5 ng/mL, *n* = 29 vs., 297.3 ± 216.8 ng/mL; *n* = 16, *p* = 0.002). Mean chemerin levels were not different between UC exacerbation and remission group (606.5 ± 234.0 ng/mL, *n* = 19 vs., 485.5 ± 182.4 ng/mL, *n* = 13; *p* = 0.128).

**Figure 1:**
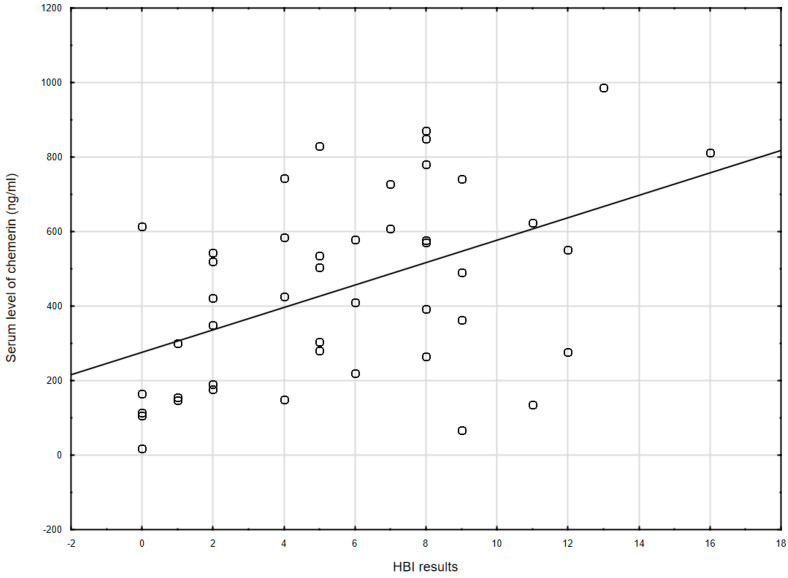
The correlation between chemerin concentration and the Harvey–Bradshaw index results (r = 0.478, *p* = 0.001) in CD patients. HBI: Harvey–Bradshaw index.

Chemerin levels correlated with the clinical severity of CD as assessed by HBI (r = 0.478, *p* = 0.001; Figure 1), but not with the severity of UC by PMS scale (r = 0.035, *p* = 0.851). Besides this, among all IBD patients, no correlation was seen between chemerin levels and BMI (r = 0.030, *p* = 0.788).

As many studies noted a correlation between BMI and chemerin, we have created a new parameter: chemerin/BMI (ng·m^2^/mL·kg). This parameter was higher in IBD patients with exacerbation than in remission (*p* = 0.003; Table 1).

**Figure 2:**
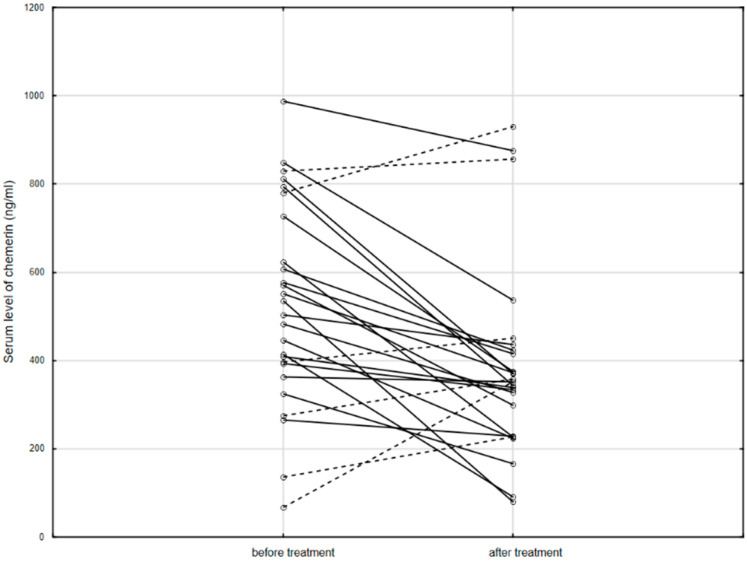
The serum level of chemerin in patients before and after anti-TNF therapy. Solid line: decreased concentration; dashed line: increased concentration.

Furthermore, serum chemerin levels significantly decreased after 14 weeks of biological treatment (519.6, IQR: 393.2–727.0 ng/mL vs. 351.5, IQR: 229.3–424.2 ng/mL; *p* = 0.002; Figure 2).

After anti-TNF therapy, 20 patients achieved clinical remission, while 6 patients still had active disease. There was no difference in the chemerin levels measured before treatment in these patient subgroups (532.2 ± 166.0 mg/mL vs. 512.7 ± 398.4 ng/mL; *p* = 0.860). However, after treatment, patients in remission had significantly lower levels of chemerin than patients with exacerbation (319.8 ± 118.7 ng/mL vs. 599.7 ± 318.9 ng/mL; *p* = 0.003). Additionally, the ratio of serum levels of chemerin measured before and after anti-TNF treatment was higher in patients, who reached remission after 14 weeks of therapy compared to the group in which the disease was still active (1.53 IQR: 1.19–2.09, *n* = 20 vs. 0.80 IQR: 0.60–0.97, *n* = 6; *p* < 0.001).

No effect of other accompanying chronic diseases, treatment with immunomodulators, or history of abdominal surgery on chemerin levels was observed. Patients treated with anti-TNF before therapy had similar levels of chemerin compared to the remaining IBD group. However, patients treated with steroids revealed higher concentrations of chemerin compared to patients who did not receive this treatment (*p* = 0.049, Table 2).

## 4. Discussion

Our results confirm the role of chemerin in the inflammatory process in IBD. We have shown significantly increased levels of chemerin in IBD patients compared to HCs. These levels were also higher in patients with active disease compared to remission, and also after adjustment for BMI. Additionally, we observed a positive correlation between CD severity and serum level of chemerin.

Until now there were only two studies evaluating chemerin levels in animal models of colitis induced by the administration of a 4% DSS solution. In one study, it was shown that chemerin levels increased after DSS administration, but the injection of exogenous chemerin did not augment further colonic inflammation [24]. In addition, in the same study, ChemR23 knockout mice compared to wild-type mice showed delayed weight loss as well as colonic inflammation; however, they were not protected against the progression of the disease. The foregoing suggests that chemerin may be involved both in initiation and resolution of inflammation [24]. In contrast to this study, Lin et al. demonstrated in a mouse model of colitis induced by DSS that the exogenous injection of chemerin caused significant weight loss, which was partially mediated by the effect of chemerin on insulin levels [30]. Additionally, chemerin aggravated the severity of colitis by causing extensive damage to the intestinal mucosa and increased pro-inflammatory cytokine levels in colon cells, including IL-6, TNF, and IFN-γ [30]. Overall, these studies demonstrated the pro-inflammatory role of chemerin in colitis and also that it may contribute to the pathogenesis of IBD.

To date, three studies have assessed the concentration of chemerin in IBD patients. Weigert et al. showed, similarly to our results, that chemerin concentration was increased in CD and UC patients compared to HCs [18]. In their study, chemerin levels were higher in CD than UC in men only. Similarly, as in our study, the circulating chemerin levels in CD and healthy subjects were not different. Increased concentrations in disease exacerbation were also observed [18]. Interestingly, UC male patients with active disease had higher chemerin concentrations than patients in remission, opposite to the CD group [18]. Among our patients, there were no differences in the concentration of chemerin between the sexes. Additionally, CD patients with exacerbation had higher serum levels of chemerin compared to those in remission, while in the UC group, the difference did not reach statistical significance. In Weigert’s study, four patients treated with glucocorticoids were mistakenly classified into the remission group, which, however, did not affect the obtained results [18]. In our study, each patient treated with glucocorticoids, regardless of the severity of clinical symptoms, was classified as the exacerbation group.

Terzoudis et al. also observed increased concentrations of chemerin in IBD patients compared to HCs [19]. However, they did not investigate the effect of disease severity on chemerin levels. Moreover, the age, BMI, and sex distributions among HCs were not specified, which seems important, due to the variability of chemerin depending on age, sex, and BMI. In another study, Waluga et al. did not find a difference between IBD and HC [20]; however, it should be noted that there was s considerable difference in age between their groups with IBD and HC [20].

We also observed a positive correlation between the clinical severity of CD and the concentration of chemerin. This confirms the importance of chemerin in the course of IBD. Interestingly, such a correlation was not observed among UC patients. Only Waluga et al. investigated the correlation between clinical severity in the entire IBD group and the concentration of chemerin, which was close to statistical significance [20]. We also noticed that the levels of chemerin were higher in the UC than in the CD group—this may be explained by the suggestion from the Dranse et al. study that local chemerin production in the cecum and colon, which is primary affected in UC, has critical biologic relevance in IBD [24]. Another explanation for this result may be that in an in vitro study performed on fibroblasts from RA patients, chemerin was found to induce TLR4 expression, which is increased in UC but not in CD [31,32].

The differences may be also due to changes in chemerin concentrations between the exacerbation and remission of disease in CD, but not in UC. Additionally, the division of patients was based on clinical scales, not an endoscopy or calprotectin test, which is a significant limitation of this study.

To the best of our knowledge, there have been no studies on the effect of anti-TNF treatment and the level of chemerin in patients with IBD. Similar studies have been conducted in other immunological diseases; in rheumatoid arthritis, ADA treatment resulted in reductions in plasma levels of chemerin, correlated with decreases in IL-6 and macrophage migration inhibitory factor concentrations [29]. Two studies which included patients suffering from psoriasis, demonstrated significantly reduced levels of chemerin after treatment with IFX [33,34]. The reason for the decrease of serum chemerin after biologic therapy is not known—it seems that chemerin concentrations are associated with the clinical conditions of patients. In other diseases (such as rheumatoid arthritis or diabetes mellitus), chemerin levels positively correlated with many pro-inflammatory factors, including TNF [29,35]. In fibroblast-like synoviocytes, it has been observed that TNF induces chemerin secretion; this may be the mechanism in which TNF blockade reduces the production of chemerin [36]. We noticed that after 14 weeks of therapy, the mean level of chemerin in patients treated with anti-TNF agents was decreased. Besides this, a greater difference between pre-treatment and post-treatment chemerin levels in patients who responded to therapy, i.e., achieved steroid-free clinical remission, was observed. This suggests that chemerin may be an indicator of ongoing inflammation in IBD, which requires confirmation in a larger group of patients treated by anti-TNF.

Additionally, patients treated with glucocorticosteroids had higher concentrations of chemerin than steroid-free patients. In line with this, Weigert et al. also observed increased levels of chemerin in patients treated with glucocorticoids [18]. The interaction of chemerin with glucocorticosteroids is not fully understood. One interesting study found that chemerin negatively correlated with luteinizing hormone—which is responsible for the production of endogenous steroids such as progesterone, testosterone, and estrogen [37,38]. However, it cannot be excluded that high concentrations of chemerin in patients treated with glucocorticoids may be due to the fact that they are administered during disease exacerbation, when chemerin levels are elevated. Interestingly, in our study, chemerin did not correlate with BMI, which contradicts the results of other authors [39,40].

Moreover, it seems that the above-discussed results regarding the effect of different treatments on chemerin levels may be a result of disease activity and may be useful in disease and treatment results monitoring. More detailed studies evaluating local chemerin in the gastrointestinal tract are necessary for understanding the role of high chemerin levels in IBD.

## 5. Conclusions

In conclusion, chemerin positively correlated with the clinical severity of IBD, and its level decreased after anti-TNF treatment. As shown in the present study, as well as in others, chemerin may be an indicator of clinical activity of IBD—CD in particular—as well as being useful in anti-TNF treatment monitoring. It would be useful to further compare the clinical value of chemerin with other known markers of IBD severity—in particular, with calprotectin. Additionally, future research should focus on the molecular basis of the relationship between chemerin levels and responses to anti-TNF therapy.

## Figures and Tables

**Table 1:** General characteristics of the study participants and the results of serum chemerin concentrations.

Parameters	IBD Group			HC (*n* = 42)	*p*
	All (*n* = 77)	Exacerbation (*n* = 48)	Remission (*n* = 29)		
Age (IQR)	35 (29–41)	35 (30–41)	36 (26–41)	31 (25–44)	^1^ 0.557 ^2^ 0.468 ^3^ 0.350
N women (%)	39 (50.6)	24 (50.0)	15 (51.7)	19 (45.2)	^1^ 0.527 ^2^ 0.955 ^3^ 0.583
BMI (kg/m^2^; IQR)	23.2 (20.2–256)	23.2 (20.6–26.1)	23.2 (19.7–25.2)	24.0 (21.4–26.4)	^1^ 0.418 ^2^ 0.920 ^3^ 0.525
HBI	5.7 (±4.0)	7.9 (±3.1)	1.5 (IQR:0.0–3.0)	N.A	^1^ N.A ^2^ <0.001 ^3^ N.A
PMS	2.5 (IQR:1.0–4.0)	3.9 (±1.7)	0.8 (IQR:0.0–1.0)	N.A	^1^ N.A ^2^ <0.001 ^3^ N.A
N smoking (%)	21 (27.3)	13 (27.1)	8 (27.6)	8 (19.0)	^1^ 0.318 ^2^ 0.962 ^3^ 0.369
N Steroids (%)	25 (32.5)	25 (52.1)	0 (0.0)	N.A	N.A
N Azathioprine (%)	27 (35.1)	14 (29.2)	13 (44.8)	N.A	^1^ N.A ^2^ 0.163 ^3^ N.A
N with other chronic diseases (%)	23 (29.9)	16 (33.3)	7 (24.1)	N.A	^1^ N.A ^2^ 0.393 ^3^ N.A
Chemerin (ng/mL)	492.3 (±244.3)	559.1 (±235.6)	381.7 (±220.2)	404.5 (±194.2)	^1^ 0.047 ^2^ 0.002 ^3^ 0.001
Chemerin/BMI (ng·m^2^/mL·kg)	21.4 (±11.0)	24.2 (±10.6)	16.7 (±10.1)	17.6 (±9.1)	^1^ 0.057 ^2^ 0.003 ^3^ 0.002

^1^ All vs. HC ^2^ exacerbation vs. remission ^3^ exacerbation vs. HC; The following statistic tests between variables were used: with normal distribution (mean with standard deviation)—*t*-test; with non-normally distributed (median with IQR)—Mann–Whitney U test; nominal variables—χ^2^ test. Abbreviations: BMI: body mass index, HBI: Harvey–Bradshaw Index, HC: healthy control, IQR: inter quartile range, IBD: inflammatory bowel disease, N: number; N.A.: not applicable; PMS: Partial Mayo Score.

**Table 2:** Relationship between the selected clinical parameters and the concentration of chemerin in the IBD patients’ blood serum.

Parameters		Chemerin (ng/mL)	*p*
Steroids *	Yes (*n* = 25) No (*n* = 52)	624.0 (IQR: 362.4–793.6) 454.0 (IQR: 288.3–598.9)	0.049
Azathioprine *	Yes (*n* = 27) No (*n* = 50)	445.7 (IQR: 264.7–613.5) 490.6 (IQR: 303.0–742.5)	0.265
Anti-TNF **	Yes (*n* = 26) No (*n* = 51)	351.5 (±229.3–424.2) 474.3 (±251.7)	0.154
History of surgery	Yes (*n* = 19) No (*n* = 58)	455.8 (±270.9) 504.3 (±236.4)	0.457
Other chronic diseases	Yes (*n* = 23) No (*n* = 53)	514.0 (±258.4) 483.1 (±240.0)	0.615
Fistulas in CD	Yes (*n* = 7) No (*n* = 38)	615.5 (±267.4) 414.8 (±240.5)	0.052
Smoking	Yes (*n* = 13) No (*n* = 64)	529.3 (±214.3) 484.8 (±250.8)	0.553

* Blood was drawn during treatment; ** Blood was drawn after 14 weeks treatment with anti-TNF; CD: Crohn’s disease; IQR: interquartile range; TNF: tumor necrosis factor.

## Data Availability

The data presented in this study are available on request from the corresponding author.
